# Supplementary material for: Baseline Characteristics of Weight-Loss Success in a Personalized Nutrition Intervention: A Secondary Analysis
Source: Nutrients. 2025 Jun 30;17(13):2178. doi: 10.3390/nu17132178 (PMC12251776; doi:10.3390/nu17132178)
Supplement: Supplementary file 1 [file nutrients-17-02178-s001.zip › nutrients-3726897-supplementary.pdf]

Supplementary Material

Variable importance ranking scores were calculated for the combined, Standardized, and Personalized arms. The variable importance ranking score is the contribution of each predictor variable to the model's predictive performance. The raw, unnormalized scores were normalized by dividing each variable's importance by the sum of the importance scores across all variables, ensuring that the scores are scaled relative to one another. Therefore, the normalized importance scores in *sTable 1-3* reflect the proportion of the total importance attributed to each variable. A higher score indicates that the variable has more influence on the model.

Table S1: Variable ranking importance in the combined arms.

Combined Arms	
**Variable**	**Importance**
WEL (score)	21.4
Age (year)	12.1
BMI (kg/m^2)	9.84
Metformin use	7.07
$TAR_{>140}$ (%)	4.96
≥Bachelor's Degree	2.50
Race, African American	1.93
Race, Other	0.89
Sex, Female	0.00
Hispanic	0.00

Importance is normalized with a total equal to 1. The formula is [importance/sum (importance)*100]

Table S2: Variable ranking importance in the Standardized arm.

Standardized Arm	
**Variable**	**Importance**
BMI (kg/m^2)	15.9
Age (year)	10.2
WEL (score)	9.27
$TAR_{>140}$ (%)	6.89
Metformin use	3.36
≥Bachelor's Degree	3.15
Sex, Female	2.10

≥$75,000 per yr	0.77
Race, African American	0.00
Race, Other	0.00

Importance is normalized with a total equal to 1. The formula is [importance/sum (importance)*100]

Table S3: Variable ranking importance in the Personalized arm.

**Personalized Arm**	
**Variable**	**Importance**
WEL (score)	19.4
BMI (kg/m^2)	14.1
Age (year)	12.6
$TAR_{>140}$ (%)	12.0
Sex, Female	1.96
Race, African American	1.35
≥$75,000 per yr	0.21
Race, Other	0.00
Hispanic	0.00
≥Bachelor's Degree	0.00

Importance is normalized with a total equal to 1. The formula is [importance/sum (importance)*100]
